# An Improved Method of Determining Human Population Distribution Based on Luojia 1-01 Nighttime Light Imagery and Road Network Data—A Case Study of the City of Shenzhen

**DOI:** 10.3390/s20185032

**Published:** 2020-09-04

**Authors:** Qiang Zhou, Yuanmao Zheng, Jinyuan Shao, Yinglun Lin, Haowei Wang

**Affiliations:** 1 Key Lab of Urban Environment and Health, Institute of Urban Environment, Chinese Academy of Sciences, Xiamen 361021, China; qzhou@iue.ac.cn (Q.Z.); yuanmaozheng@iue.ac.cn (Y.Z.); jyshao@iue.ac.cn (J.S.); yllin@iue.ac.cn (Y.L.); 2 University of Chinese Academy of Sciences, Beijing 100049, China

**Keywords:** urban population distribution, road network density, nighttime light imagery, Luojia 1-01 data, human settlement index

## Abstract

Previously published studies on population distribution were based on the provincial level, while the number of urban-level studies is more limited. In addition, the rough spatial resolution of traditional nighttime light (NTL) data has limited their fine application in current small-scale population distribution research. For the purpose of studying the spatial distribution of populations at the urban scale, we proposed a new index (i.e., the road network adjusted human settlement index, RNAHSI) by integrating Luojia 1-01 (LJ 1-01) NTL data, the enhanced vegetation index (EVI), and road network density (RND) data based on population density relationships to depict the spatial distribution of urban human settlements. The RNAHSI updated the high-resolution NTL data and combined the RND data on the basis of human settlement index (HSI) data to refine the spatial pattern of urban population distribution. The results indicated that the mean relative error (MRE) between the population estimation data based on the RNAHSI and the demographic data was 34.80%, which was lower than that in the HSI and WorldPop dataset. This index is suitable primarily for the study of urban population distribution, as the RNAHSI can clearly highlight human activities in areas with dense urban road networks and can refine the spatial heterogeneity of impervious areas. In addition, we also drew a population density map of the city of Shenzhen with a 100 m spatial resolution for 2018 based on the RNAHSI, which has great reference significance for urban management and urban resource allocation.

## 1. Introduction

Population data are not only the basic data that reflect the social and economic situation of a country or region, but are also some of the most important basic data in social and geographical research [1]. Population data have been extensively used for social resource allocation, environmental protection, and city planning [2]. However, existing population data are often collected step-by-step with administrative divisions as units, with a long update cycle and low spatial and temporal resolution. The above problems can be effectively solved by the spatialization of population data, which is one of the most important methods to realize the coupling of population and other socio-economic, resource, and environmental data. This carries important implications to enhance the comprehensive management capacity of populations, resources, and the environment [3]. 

In the past few decades, the rapid development of geospatial information technology has provided the possibility of multi-scale population density estimation. Scholars have explored a variety of population density estimation methods using both multi-source remote sensing and auxiliary data [4,5,6,7]. Lo et al. [8] divided the methods of population density estimation into four categories: the collection of individual residential units, the measurement of urbanized land area, the estimation of land use classification, and automatic analysis of imagery based on spectral features. Following on from the basic principles and development process of population spatialization, Bai et al. [9] divided the methods of population spatialization into three categories: models of urban geography population density, spatial interpolation, and statistical models based on remote sensing and geographic information systems (GIS). Among them, the model of urban geography population density was not directly utilized for population spatialization, but more to research the regulation of urban population distribution [9]. The uncertainty of spatial interpolation was exceedingly high, while the above difficulties were overcome with the statistical modeling method based on remote sensing and GIS, which is the focus of the current research.

Alongside the rise in popularity of high-resolution remote sensing data, the spatialization method based on pixel features has become an important means of population spatialization [9], Min et al. [10] used cellular automata and multi-agent methods, combined with land classification and location information of public facilities, to study the spatial distribution of the population in Beijing. The core of cellular automata is how to define transition rules, which can be represented in many forms [11]. However, most of transition rules in the above study are defined statically by several equations; as a result, the internal structure of the city has not been well described. Nowadays the modeling of auxiliary data has gradually developed from using land use data to using building information and social media data. In commonly used remote sensing images, nighttime light (NTL) data have attracted wide industrial interest, because they can acutely detect light of different intensities [12], and can quickly and comprehensively characterize the intensity and breadth of the spatial distribution of a population using fewer data sources. 

Lu et al. [13] proposed the human settlement index (HSI) to research the population distribution of Zhejiang Province in China by using Defense Meteorological Satellite Program Operational Linescan System (DMSP-OLS) and normalized difference vegetation index (NDVI) data. Yang et al. [14] updated the enhanced vegetation index (EVI) data and proposed the elevation-adjusted HSI (EAHSI) combined with elevation data on the basis of the HSI. However, the application of DMSP-OLS data is less common, as these data are only available until 2013 and have low spatial resolution (1 km). The emergence of Suomi National Polar-Orbiting Partnership Visible Infrared Imaging Radiometer Suite (NPP-VIIRS) data effectively overcomes the shortcomings of DMSP-OLS data in spatio-temporal resolution [15], and such data are more suitable for the study of human social and economic activities [16]. 

Based on NPP-VIIRS and land use data, Hu et al. [17] established a stepwise regression model to study the population spatialization in the Sichuan–Chongqing area of China. He et al. [18] used NPP-VIIRS NTL data to research the population spatialization of Beijing using the random forest method. The early release of NPP-VIIRS data is of great help for the simulation of population distribution in a long time series. In addition, the strip width of 3000 km provides great convenience for the study of population spatialization in large areas; however, the resolution of 500 m still has a few limitations in the fine-scale study of population spatialization. At the same time, multi-source remote sensing images combined with auxiliary data have been widely used in population spatial distribution, including several famous population datasets, such as the LandScan (1 km) [19], Gridded Population of the World (1 km) [20], and the WorldPop (1 km and 100 m) [21]. However, the resolution of such remote sensing data is still insufficient to reflect actual human activities in urban areas [22], which could greatly limit the accuracy of these population datasets within a small range [23].

With the rapid development of remote sensing technology, the world’s first professional luminous remote sensing satellite designed by Wuhan University, Luojia 1-01 (LJ 1-01), was successfully launched in June 2018. Compared to NPP-VIIRS data, LJ 1-01 data have a significant improvement in spatial resolution and quantitative level, which provide a more refined data source for small-scale population distribution simulations [24,25,26]. These incomparable advantages make it an important supplement to NTL data products; however, its current application in the study of population spatialization (especially at the urban scale) is almost non-existent. Researchers have shown that the majority of the world’s population is concentrated in low-altitude plains [27], and the two types of HSI proposed in previous research would be not applicable to study the urban population distribution in such low-altitude areas. Road network density (RND) is an important variable for simulating urban population distribution, as the road grid bureau is the spatial structure of urban population distribution [28]. Considering the importance of RND in simulating social and economic parameters (such as population and gross domestic product), there are few studies utilizing RND for correction to improve the HSI.

Here, we integrated road network data with multi-source remote sensing data to further improve the accuracy of the population modeling. The result was compared with a widely used global population product. This study has introduced the field of high-resolution population modeling by utilizing an innovative combination of remote sensing and traffic road network data to refine population distribution.

In this paper, multi-source data such as LJ 1-01 NTL imagery, the EVI, and road network data were used to monitor human activities and to simulate the population distribution in Shenzhen. In order to achieve this goal, we proposed the road network adjusted human settlement index (RNAHSI) to improve the applicability of the HSI in city-scale population simulation. Subsequently, we studied the correlation between the demographic data and the sum of the RNAHSI at the township level. Based on the high correlation between the RNAHSI and the demographic data, a high-resolution (100 × 100 m) population density raster map of Shenzhen was produced. The accuracy of this method was better than that of the HSI and WorldPop dataset in terms of experimental accuracy verification. The results further indicate that this method has great potential in the population simulation of urban or even smaller-scale populations.

## 2. Study Area and Data

### 2.1. Study Area

Located in the south of Guangdong Province, Shenzhen is one of the four central cities in the Guangdong–Hong Kong–Macao Greater Bay Area [29] and is both a national logistics hub and an international integrated transport hub. Shenzhen is also a port city with the largest number of ports, the largest number of entry–exit personnel, and the largest traffic flow [30]. By the end of 2018, the city had ten districts under its jurisdiction, with a total area of 1997.47 km^2^, a built-up area of 927.96 km^2^, a permanent population of 13,026,600 people, and an urban population of 13,026,600 people. The urbanization rate was 100%, making it the first fully urbanized city in China [31].

### 2.2. Data

#### 2.2.1. High-Resolution NTL Data

**Figure 1:**
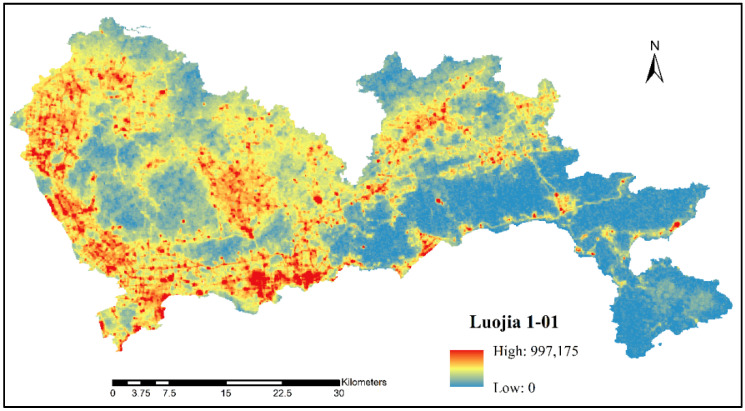
Luojia 1-01 (LJ 1-01) nighttime light (NTL) imagery in Shenzhen for the year 2018.

The NTL data in this study were obtained from LJ 1-01 on 24 November 2018 (Figure 1). The satellite was developed by Wuhan University, with a resolution of 130 m and a width of 250 km. This new kind of NTL data source provides new ideas for the investigation of population spatialization in city areas, which can not only improve the precision of urban population density mapping but can also better reveal the detailed characteristics of urban population distribution. The data are free to download from the High-Resolution Earth Observation System of the Hubei Data and Application Center website [32].

#### 2.2.2. Road Network Data

Compared to traditional GIS, OpenStreetMap (OSM) data [33] have the advantages of fast updating, reflecting the real situation, and low cost [34]. In addition to being navigation data, OSM data also provide a new idea for the collection and updating of urban road network basic data [35]. A large number of studies have shown that OSM data perform better for detail in urban areas, while the accuracy of the attributes in rural areas is poor [36]. Therefore, we selected OSM data as the experimental data of the traffic network.

#### 2.2.3. Auxiliary Data

The population statistical data at the township level were derived from the 2018 China County Statistical Yearbook [37] and the 2018 Statistical Yearbook of Shenzhen and various other districts [38]. The administrative unit boundaries were obtained from the Guangdong Geographic Information Public Service Platform [39]. For the accuracy assessment, we also used the WorldPop dataset for the year 2018 [40] with a spatial resolution of 100 m, which is one of the most accurate datasets for the estimation of population distribution [9].

The EVI is based on MOD13Q1-EVI data, which were acquired from the Data Information Service Center of National Aeronautics and Space Administration [41]. The EVI has a spatial resolution of 250 m and a time resolution of 16 days, and it spans the year 2018. This data product has undergone solar radiation correction, atmospheric correction, and aerosol, water, cloud, etc. processing, which minimizes the impact of atmospheric and soil reflectance. Compared to the NDVI, these data reduce the influence of atmospheric and soil background values, and are more suitable for analyzing vegetation changes in sparse vegetation areas, such as cities and towns [42]. Since the data are in 16-day intervals, we downloaded a total of 23 images for the year 2018.

## 3. Methodology

### 3.1. Data Preprocessing

We first re-projected the LJ 1-01 NTL data, the EVI data, and the WorldPop dataset to the Universal Transverse Mercator (UTM) system. Subsequently, a bilinear interpolation algorithm was utilized to resample the LJ 1-01 and EVI data for unifying the spatial resolution to 100 m, which is the same as that of the WorldPop dataset. According to [43], the LJ 1-01 NTL imagery was registered with high-precision Google Map imagery, and then radiometric correction was performed. The digital number (DN) value was converted into the radiant brightness (RB) value to participate in the subsequent calculation. The radiance conversion formula of LJ 1-01 NTL is as follows [44]:

(1) $$L=10^{-10}\times DN^{\frac{2}{3}}$$

where *L* represents the RB values with the unit W/(m^2^∙sr∙μm), and *DN* represents the *DN* values for a pixel.

**Figure 2:**
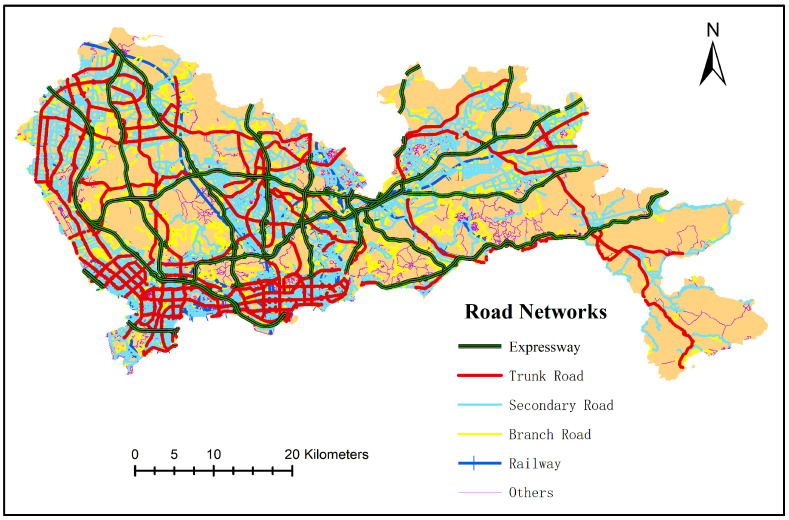
The distribution of the road network in Shenzhen.

According to the classification and interpretation of the OSM road grade in Wikipedia [45], the road network data of the city of Shenzhen were extracted and divided into six categories: expressways, trunk roads, secondary trunk roads, branch roads, railways (including subways), and others (including cycle lanes, footpaths, and residential streets). The distribution of the road network is shown in Figure 2. As many roads were two-way roads, the subsequent calculation of the road network length would need to be repeated, which would affect the accuracy of the results. Therefore, we used ArcGIS software to extract the center line of the double line elements in the road network.

In order to eliminate the impact of clouds, the maximum algorithm was utilized to generate a new EVI composite image from the 23 EVI images taken in 2018 [14].

### 3.2. Generating a HSI Image

An HSI image for Shenzhen for the year 2018 was produced from the *EVI* composite image and LJ 1-01 data, as expressed in Equation (2):

(2) $$\mathrm{HSI}\;=\frac{1-EVI_{max}+LJ_{nor}}{1-LJ_{nor}+EVI_{max}+LJ_{nor}\times EVI_{max}}$$

where *LJ_nor_* denotes the normalized RB values of the LJ 1-01 NTL imagery, which was calculated as follows:

(3) $$LJ_{nor}=\frac{LJ-LJ_{min}}{LJ_{max}-LJ_{min}}$$

where *LJ_max_* and *LJ_min_*, respectively, denote the maximum and minimum RB values of the LJ 1-01 NTL imagery in Shenzhen.

### 3.3. Generating an RND Layer

**Figure 3:**
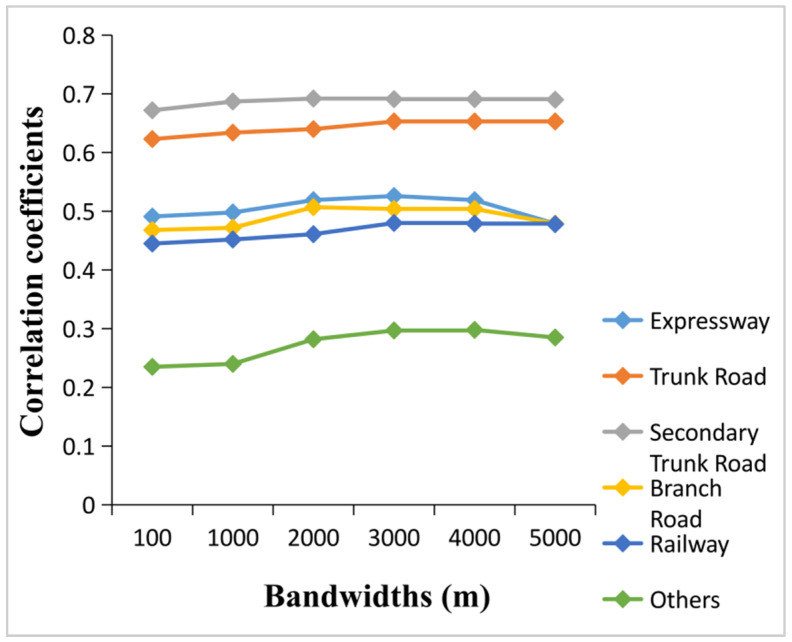
Line chart between the correlation coefficients and bandwidths.

The kernel density estimation (KDE) method primarily estimates the density of a point or line pattern with the help of a moving cell (equivalent to a window) [46,47]. In KDE estimation, the determination or selection of bandwidth has a great influence on the calculation results. With an increase in bandwidth, the change of the point density in space is smoother but masks the structure of the density; when the bandwidth decreases, the point density becomes abrupt and uneven [46]. In specific application events, the value of the bandwidth is flexible, and experiments must be conducted according to the different bandwidth values. A default bandwidth is automatically generated, which is obtained by dividing the minimum bandwidth or length in the analyzed data layer by 30 in the ArcGis KDE. As the automatically generated bandwidth of each type of road network was different, we tested different bandwidths between 100 and 5000 m at an interval of 100 m according to the linear correlation between the total kernel density of each type of road network and the population in each township. As shown in Figure 3, the fluctuation of the correlation coefficient of all kinds of road networks was the smallest when the bandwidth was between 2000 and 4000 m. Ultimately, we chose 3000 m as the bandwidth for calculating the KDE of each type of road network, as their correlation coefficients with population were relatively high and stable.

Principal component analysis (PCA) changes the relevant variables into several unrelated comprehensive index variables through the method of variable change in order to achieve dimension reduction of the dataset and to simplify the problem [48]. The sum of each type of RND and the demographic data at the township level were analyzed by PCA, and their weights were calculated to generate a composite RND layer.

### 3.4. Accuracy Assessment

To emphasize that the introduction of RND data by the HSI could notably increase the exactitude of the population density maps, we compared the RNAHSI to the HSI and the WorldPop dataset. The population estimation data of the three were summarized at the township level and compared to the demographic data to assess the accuracy. For these three methods, the summary statistics were calculated, including the mean relative error (MRE) and the root mean square error divided by the mean township population count (%RMSE). The MRE can be utilized to quantitatively reflect the mean oscillation amplitude of the deviation between the estimated and measured values. Meanwhile, the %RMSE can be used to measure the deviation between the estimated and actual values, can well reflect the precision of the simulation, and can also evaluate the predictive ability of each model. These metrics are calculated as:

(4) $$\mathrm{MRE}=\frac{1}{n}\overset{n}{\underset{i=1}{\sum }}\frac{\left|PE_{i}-P_{i}\right|}{P_{i}}$$

(5) $$\%\mathrm{RMSE}=\frac{\sqrt{\frac{\sum_{i=1}^{n}{\left(PE_{i}-P_{i}\right)}^{2}}{n}}}{M_{pop}}$$

where *PE_i_* represents the estimated population of the ith town, *P_i_* represents the actual population of the ith town, *n* is the number of towns, and *M_pop_* is the mean township population.

## 4. Results

### 4.1. Road Network Density

**Figure 4:**
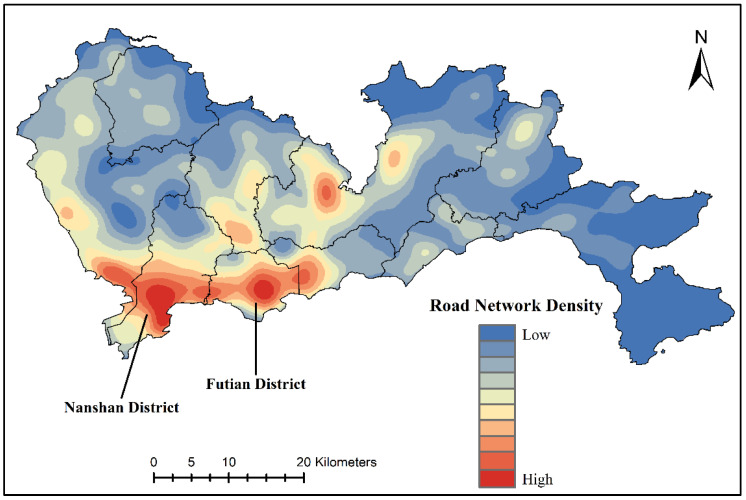
The kernel density composite image of the road networks.

The weights of the expressways, trunk roads, secondary trunk roads, branch roads, railways and others were calculated as 0.2, 0.15, 0.08, 0.15, 0.21, and 0.2, respectively, through PCA. A composite RND image (Figure 4) was generated by overlaying six road network kernel density maps with their respective weights. The majority of the road networks in Shenzhen were concentrated in the southwest, particularly in Nanshan District and Futian District, and the road networks in the north and southeast were the sparsest. The distribution of the road network in Shenzhen was generally consistent with the NTL data.

### 4.2. The Calculation of the RNAHSI

**Figure 5:**
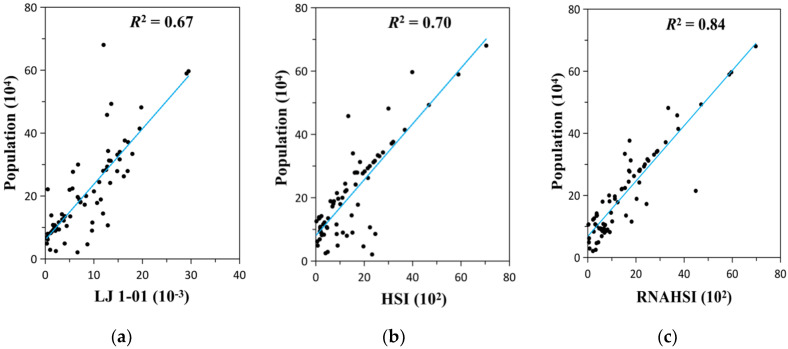
Scatterplots between the actual population at the township level and (**a**) the cumulative radiant brightness (RB) values of the LJ 1-01 NTL data, (**b**) the cumulative human settlement index (HSI), and (**c**) the cumulative RNAHSI at the township level in Shenzhen for the year 2018.

As shown in Figure 5a, by observing the scatterplot between the cumulative RB values of LJ 1-01 NTL data and the total population at the township level in Shenzhen, the two had a linear relationship with a coefficient of determination (R^2^) equal to 0.67, which was not very strong. Considering the existence of underground commercial streets and underground traffic in Shenzhen, LJ 1-01 could not detect the NTL in these areas, resulting in the loss of the NTL DN value, which finally led to a low correlation between it and the population. The linear relationship between the HSI and the total population at the township level is presented in Figure 5b, where the R^2^ is equal to 0.70. Compared to the former result, this fitting result did not improve much, mainly because the above-mentioned problems remained unsolved. Therefore, HSI could be unsuitable for the prediction of urban population distribution.

**Figure 6:**
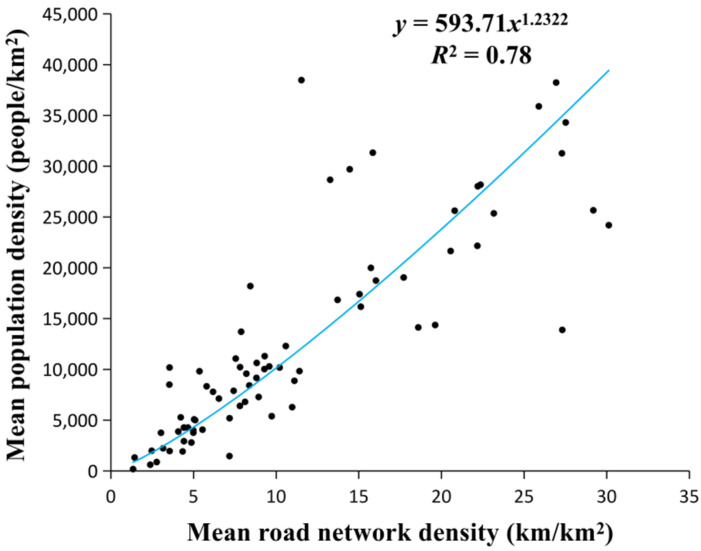
Regression analysis between the mean road network density (RND) and the mean population density in Shenzhen for the year 2018.

Based on the above analysis, to estimate the urban population distribution more accurately, we corrected the HSI using road networks to solve the problem of population underestimation in areas with dense road networks. The mean population density (i.e., the ratio of the total population to the area at the township level) and the mean *RND* (i.e., the ratio of the total *RND* to the area at the township level) were regressed. The results illustrate that they were distributed as power functions and highly correlated, with an R^2^ of 0.78 (Figure 6). Based on this, we took the *RND* as an important factor to improve the urban HSI, to correct the road networks of the HSI, and to obtain the RNAHSI. The improved formula is as follows:

(6) $$\mathrm{RNAHSI}=\frac{1-EVI_{max}+LJ_{nor}}{1-LJ_{nor}+EVI_{max}+LJ_{nor}\times EVI_{max}}\times RND^{1.2322}$$

After the correction of road networks, the HSI converged to the regression straight line more than before (Figure 5c), and the R^2^ increased from 0.70 to 0.84. This indicates that the model is reasonable at improving the HSI to estimate urban populations.

### 4.3. Population Density

**Figure 7:**
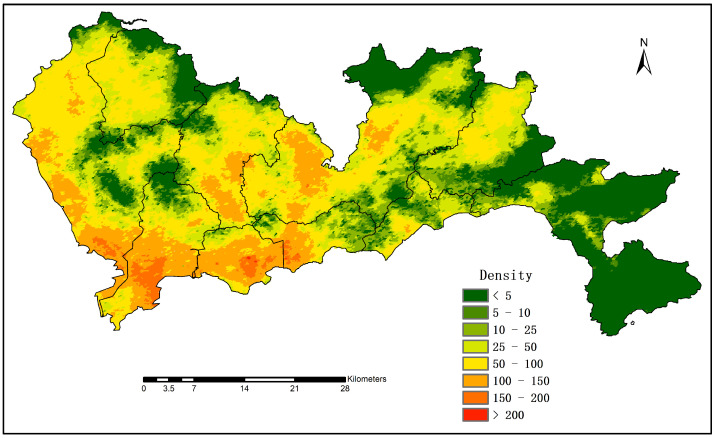
Population density map of Shenzhen in 2018 (units: individuals/0.01 km^2^).

As there was a strong linear relationship between the RNAHSI and the population at the township level, we produced a population distribution map of Shenzhen on this basis. To eliminate the overall bias of the RNAHSI, the total actual population was divided by the estimated population, and this ratio (0.82 in this study) was utilized as a correction factor. Figure 7 shows the population density raster map of Shenzhen for the year 2018 with a spatial resolution of 100 m. The population of Shenzhen was principally concentrated in the central and western regions, and Dapeng New District in the east was the most sparsely populated area. The southern part of Bao’an District, Nanshan District, Futian District, and the west of Luohu District were the most densely populated areas due to their proximity to Hong Kong. Due to the high resolution of the LJ 1-01 NTL data and the correction of the road networks, the urban population distribution in this study illustrated more details and more obvious spatial heterogeneity. Urban population density maps can be generally applied for urban resource allocation, population research, decision-making, and urban emergency responses. In addition, they are also of great help in urban risk management. For example, during the COVID-19 pandemic, urban population density maps could be important databases for the government to carry out prevention and control work. We have released Shenzhen population density map at GitHub [49] to facilitate readers and those in need to use and further research.

### 4.4. Accuracy Assessment

Table 1 provides a summary of the accuracy verification of the HSI, the RNAHSI, and the WorldPop dataset. When using the HSI to estimate population, the MRE and %RMSE were 74.35% and 83.26%, respectively. The estimated population of several areas with dense road networks in Shenzhen was underestimated, as the existence of underground commercial streets and underground traffic was not considered. Although the introduction of the EVI by the HSI allowed us to distinguish the vegetation areas and impermeable areas well, there was still a large error when it was applied to urban population distribution. After combining road network data based on population density on the basis of the HSI, the MRE (34.80%) and %RMSE (42.29%) decreased significantly, and were lower than that of the WorldPop dataset (MRE = 47.36% and %RMSE = 54.15%).

**Figure 8:**
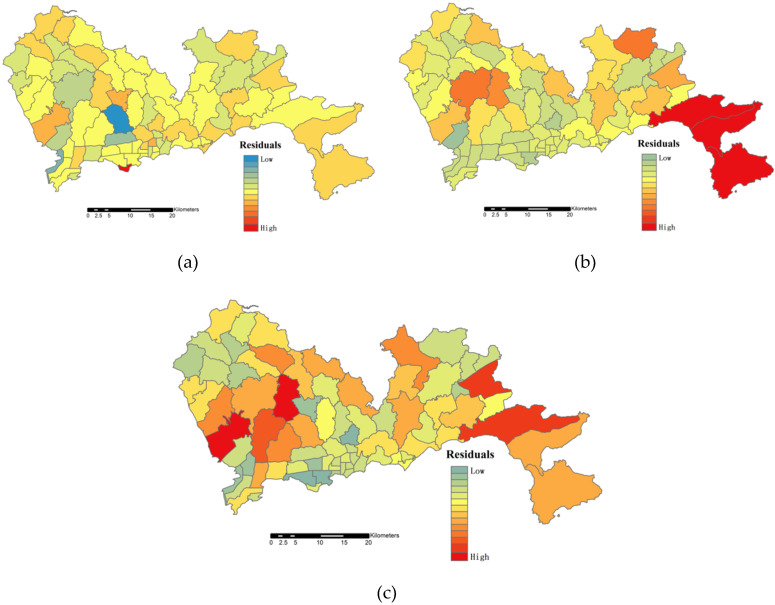
Township-level distribution of the residuals of the predicted population using (**a**) the RNAHSI, (**b**) the HSI, and (**c**) the WorldPop dataset.

We compared the residual distribution of the estimated population between the RNAHSI (Figure 8a), the HSI (Figure 8b), and the WorldPop dataset (Figure 8c). The residual value was obtained using the estimated population minus the demographic data at the township level. The red bars indicate overestimation of the population, while the blue bars indicate underestimation of the population, and the darker the color, the higher the degree of overestimation or underestimation. The population predicted by the HSI was widely underestimated in areas with high RND, which was seriously overestimated in Dapeng New District. The population was slightly underestimated in a few regions by the RNAHSI method, but the RNAHSI significantly improved the residual distribution in general, and the error was lower than that of the WorldPop dataset. 

Therefore, the RNAHSI can reduce the error of population density modeling and can improve the accuracy in complex urban road network areas. However, the estimated total population of Shenzhen remained underestimated. The demographic data were derived from the permanent resident population counted by the government and its statistical departments, excluding the floating population, while Shenzhen is a city with high population mobility, which may have led to the underestimation of the population model. In the future, the problem of population underestimation in these regions can be improved by using more detailed census data.

## 5. Discussion

The spatial distribution of the population has always been a hot research topic. Nighttime remote sensing images have been extensively applied for spatial data mining in the socio-economic field due to their unique ability to reflect human social activities. For a long time, researchers have mostly used DMSP-OLS and NPP-VIIRS NTL data to conduct studies of population simulation and spatial distribution. However, the emergence of LJ 1-01 NTL data has provided more effective data support for research with small-scale and high-precision population simulations. Previous studies have demonstrated that the fitting effect of the LJ 1-01 NTL data is better than that of NPP-VIIRS data as a whole in the simulation of population distribution, and the “blooming” effect of light is alleviated to some extent, which has a certain availability and superiority in the study of population spatialization [50]. However, LJ 1-01 NTL images could only be obtained after June 2018, resulting in the inability to synthesize the annual imagery. The utilization of annual composite imagery would be more convincing for this type of study, which could be a topic for future research. The majority of the previous studies on the HSI were based on the provincial level [11,12], while relatively few urban-level population distributions were conducted. The integration of a vegetation index (such as the NDVI or the EVI) and NTL data can well identify human settlements and impervious areas, thereby reducing the error in population estimation [51]. Although this study used LJ 1-01 NTL data, which had a high spatial resolution of 130 m, there was still some error in the population estimation of Shenzhen based on use of the HSI. 

As the population model based on the HSI failed to further refine the structure of urban impervious areas, only the buildings were taken as the main body of the impervious surface, thus ignoring the significant impact of the RND on urban population distribution [52], particularly in modern cities, such as Shenzhen, with a high RND. In the city, the communication and transportation between materials and residents rely on roads. The higher the density of the road network, the better the accessibility of the road and the more attractive it is for residents to live; at the same time, the more residents that move in, the more appropriate the road traffic will be for the travel of residents, and the density of the road network will be further improved.

Consequently, the RND data based on the relationship of population distribution combined with the HSI can effectively break through the bottleneck of urban population prediction when using the HSI. The road network data obtained from OpenStreetMap included not only nominal roads, but also commercial pedestrian streets, sidewalks, bicycle lanes, etc., which were closely related to the lives of urban residents and provided the possibility for quantifying the complex urban population distribution. Our research indeed verified that the RNAHSI can reduce errors and improve the accuracy of the results from simulating the urban population distribution. 

However, this study possesses a few shortcomings. First, since RNAHSI was established on the basis of the demographic data, which were derived from the permanent resident population counted by the government and its statistical departments, excluding the floating population, while Shenzhen is a city with high population mobility. In this way, the population predicted by this model did not include the floating population, which ultimately led to an underestimation of the predicted results. More perfect population data need to be used or developed in future research. Second, although the LJ 1-01 NTL data have a high spatial resolution and play a significant role in improving the distribution of urban population, but there are many slums or villages-in-city in the cities. For example, in this study, there are a few villages-in-cities in Shenzhen, where a large population gathers, and the intensities of NTL are relatively weak. However, the road network data cannot solve such problems, which is another reason why population predicted are underestimated by this method. Therefore, the above problems need to be solved urgently in future research, which can be achieved through the combination with social media data, building data, and so on.

Finally, this study utilized township-scale demographic data to verify the accuracy of the population spatialization results; however, reflecting the accuracy of the results on the smaller scale (smaller than the town-scale, such as communities, etc.) was difficult, which made the verification process deficient. It is mainly caused by the difficulty in obtaining the population of fine-scale units. In future research, in order to carry out more rigorous verification, the collection of more fine-scale population verification data and the establishment of a more sophisticated and reasonable accuracy verification system are necessary.

## 6. Conclusions

In this paper, to describe the spatial distribution of urban human settlements in greater detail, a new index (i.e., the RNAHSI) was proposed, integrating LJ 1-01 nighttime radiance, EVI, and RND data. For the RNAHSI, we updated the high-resolution NTL data and combined the RND data on the basis of the HSI to improve the spatial pattern of urban population distribution. The results indicated that the method well reflected the spatial distribution of urban human settlements and greatly reduced the impact of urban traffic structure on population distribution. In terms of accuracy verification, the overall error of urban population distribution simulated by the RNAHSI is lower than that of the HSI, or even lower than that of the WorldPop dataset. In summary, the RNAHSI is suitable primarily for the study of urban population distribution, as the RNAHSI can clearly highlight human activities in areas with dense urban road networks and can refine the spatial heterogeneity of impervious areas. In addition, we also drew a population density map of Shenzhen with a 100 m spatial resolution for 2018 based on the RNAHSI, which could provide great reference significance for urban management and urban resource allocation.

## Figures and Tables

**Table 1:** Accuracy comparison of the HSI, the RNAHSI, and the WorldPop dataset.

	HSI	RNAHSI	WorldPop
R^2^	0.70	0.84	0.77
MRE (%)	74.35	34.80	47.36
%RMSE	83.26	42.29	54.15

R^2^, Coefficient of determination; MRE, mean relative error; %RMSE, root mean square error divided by the mean township population count.

## References

[1] Hu H. (1983). Essays on China’s Population Distribution.

[2] Wen X., Li B., Wang W., Li S., Ou X., Zheng Y. (2006). Deposition of sandstorms in a vegetation-covered sand dune in Ejin Oasis and its characteristics. J. Geogr. Sci..

[3] Zhuo L., Chen J., Shi P., Gu Z., Fan Y., Ichinose T. (2005). Modeling population density of China in 1998 based on DMSP /OLS nighttime light image. J. Geogr..

[4] Wu S., Qiu X., Wang L. (2005). Population Estimation Methods in GIS and Remote Sensing: A Review. GIScience Remote Sens..

[5] Wu C., Murray A.T. (2007). Population Estimation Using Landsat Enhanced Thematic Mapper Imagery. Geogr. Anal..

[6] Li L., Lu D. (2016). Mapping population density distribution at multiple scales in Zhejiang Province using Landsat Thematic Mapper and census data. Int. J. Remote Sens..

[7] Li X., Chen Z., Wu J., Wang W., Qu L., Zhou S., Han X. (2017). Gridding Methods of City Permanent Population Based on Night Light Data and Spatial Regression Models. J. Geo-Inf. Sci..

[8] Lo C.P. (1986). Applied remote sensing. Geocarto Int..

[9] Bai Z., Wang J., Wang M., Gao M., Sun J. (2018). Accuracy Assessment of Multi-Source Gridded Population Distribution Datasets in China. Sustainability.

[10] Min Y., Zou Y., Wang Q., Rao P. (2019). Study of urban population spatial distribution based on CA/MAS. Mod. Surv. Mapp..

[11] Li X., Yang Q., Liu X. (2008). Discovering and evaluating urban signatures for simulating compact development using cellular automata. Landsc. Urban Plan..

[12] Elvidge C.D., Sutton P.C., Pettit D.R., Cinzano P., Small C. (2007). Overview of the Nightsat mission concept. Proceedings of the 2007 Urban Remote Sensing Joint Event, Paris, France, 11–13 April 2007.

[13] Lu D., Tian H., Zhou G., Ge H. (2008). Regional mapping of human settlements in southeastern China with multisensor remotely sensed data. Remote Sens. Environ..

[14] Yang X., Yue W., Gao D. (2013). Spatial improvement of human population distribution based on multi-sensor remote-sensing data: An input for exposure assessment. Int. J. Remote Sens..

[15] Chen Y., Zheng Z., Wu Z., Qian Q. (2019). Review and prospect of application of nighttime light remote sensing data. Adv. Earth Sci..

[16] Chen Z., Yu B., Hu Y., Huang C., Shi K., Wu J. (2015). Estimating House Vacancy Rate in Metropolitan Areas Using NPP-VIIRS Nighttime Light Composite Data. IEEE J. Sel. Top. Appl. Earth Obs. Remote Sens..

[17] Hu Y., Zhao G., Zhang Q. (2018). Spatial Distribution of Population Data Based on Nighttime Light and LUC Data in the Sichuan-Chongqing Region. J. Geo-Inf. Sci..

[18] He M., Xu Y., Li N. (2020). Population Spatialization in Beijing City Based on Machine Learning and Multisource Remote Sensing Data. Remote Sens..

[19] Cui C., Lelgemann D. (2000). On non-linear low-low SST observation equations for the determination of the geopotential based on an analytical solution. J. Geod..

[20] Doxsey-Whitfield E., MacManus K., Adamo S.B., Pistolesi L., Squires J., Borkovska O., Baptista S.R. (2015). Taking Advantage of the Improved Availability of Census Data: A First Look at the Gridded Population of the World, Version 4. Pap. Appl. Geogr..

[21] Tatem A.J. (2017). WorldPop, open data for spatial demography. Sci. Data.

[22] Wu C.-D., Lung S.-C.C. (2012). Applying GIS and fine-resolution digital terrain models to assess three-dimensional population distribution under traffic impacts. J. Expo. Sci. Environ. Epidemiol..

[23] Jia P., Qiu Y., Gaughan A.E. (2014). A fine-scale spatial population distribution on the High-resolution Gridded Population Surface and application in Alachua County, Florida. Appl. Geogr..

[24] Li X., Zhao L., Li D., Xu H. (2018). Mapping Urban Extent Using Luojia 1-01 Nighttime Light Imagery. Sensors.

[25] Jiang W., He G., Long T., Guo H., Yin R., Leng W., Liu H., Wang G. (2018). Potentiality of Using Luojia 1-01 Nighttime Light Imagery to Investigate Artificial Light Pollution. Sensors.

[26] Li F., Yan Q., Bian Z., Liu B., Wu Z. (2020). A POI and LST Adjusted NTL Urban Index for Urban Built-Up Area Extraction. Sensors.

[27] Cohen J.E., Small C. (1998). Hypsographic demography: The distribution of human population by altitude. Proc. Natl. Acad. Sci. USA.

[28] Zhao F., Sun H., Wu J., Gao Z., Liu R. (2016). Analysis of Road Network Pattern Considering Population Distribution and Central Business District. PLoS ONE.

[29] The Central Committee of the Communist Party of China The outline of Guangdong-Hong Kong-Macau Greater Bay Area’s Development Plan. http://www.gov.cn/zhengce/2019-02/18/content_5366593.htm#1.

[30] Yi M. (2012). “Twelfth Five-Year” Comprehensive Transportation System Planning. Integr. Transp..

[31] Shenzhen Bureau of Statistics, National Bureau of Statistics (2018). Shenzhen Investigation Team Statistical Bulletin of Shenzhen’s National Economic and Social Development in 2018.

[32] High-Resolution Earth Observation System of the Hubei Data and Application Center. http://59.175.109.173:8888/.

[33] OpenStreetMap. https://www.openstreetmap.org/.

[34] Luo L., Liu B., Liu X. (2017). Data Quality Assessment and Application Analysis for OpenStreetMap Road Network. Jiangxi Sci..

[35] Wang M., Li Q., Hu W., Zhou M. (2013). Quality Evaluation Method of Open Street Map Spatial Data for Public Sources. J. Wuhan Univ..

[36] Sehra S.S., Singh J., Rai H.S. (2014). A Systematic Study of OpenStreetMap Data Quality Assessment. Proceedings of the 2014 11th International Conference on Information Technology: New Generations, Las Vegas, NV, USA, 7–9 April 2014.

[37] Rural Social and Economic Investigation Department, National Bureau of Statistics (2019). China County Statistical Yearbook-2018.

[38] Statistics Bureau of Shenzhen Municipality (2019). Statistics Bureau of Shenzhen Municipality.

[39] Guangdong Geographic Information Public Service Platform. http://guangdong.tianditu.gov.cn/.

[40] Tatem A.J., Gaughan A.E., Stevens F.R., Patel N.N., Jia P., Pandey A., Linard C. (2013). Quantifying the effects of using detailed spatial demographic data on health metrics: A systematic analysis for the AfriPop, AsiaPop, and AmeriPop projects. Lancet.

[41] Data Information Service Center of National Aeronautics and Space Administration. https://ladsweb.modaps.eosdis.nasa.gov/search/.

[42] Dallimer M., Tang Z., Bibby P.R., Brindley P., Gaston K.J., Davies Z.G. (2011). Temporal changes in greenspace in a highly urbanized region. Biol. Lett..

[43] Wang L., Fan H., Wang Y. (2020). Improving population mapping using Luojia 1-01 nighttime light image and location-based social media data. Sci. Total Environ..

[44] Li X., Wang T., Zhang G., Jiang B., Jia P., Zhang Z., Zhao Y. (2019). Planar Block Adjustment for China’s Land Regions with LuoJia1-01 Nighttime Light Imagery. Remote Sens..

[45] The Classification and Interpretation of the OSM Road Grade. https://wiki.openstreetmap.org/wiki/Key:highway.

[46] Wang Y., He H. (2007). Spatial Data Analysis Method.

[47] Shi X. (2010). Selection of bandwidth type and adjustment side in kernel density estimation over inhomogeneous backgrounds. Int. J. Geogr. Inf. Sci..

[48] Demšar U., Harris P., Brunsdon C., Fotheringham A.S., McLoone S. (2013). Principal Component Analysis on Spatial Data: An Overview. Ann. Assoc. Am. Geogr..

[49] Shenzhen Population Density Map at GitHub. https://github.com/Zhouqiang709/Shenzhen-population-density-map.

[50] Zou Y., Yan Q., Huang J., Li F. (2020). Modeling the Population Density of Su-Xi-Chang Region Based on Luojia-1A Nighttime Light Image. Resour. Environ. Yangtze Basin.

[51] Roy Chowdhury P.K., Maithani S. (2010). Monitoring growth of built-up areas in indo-gangetic plain using multi-sensor remote sensing data. J. Indian Soc. Remote Sens..

[52] Tan M., Liu K., Liu L., Zhu Y., Wang D. (2017). Spatialization of population in the Pearl River Delta in 30 m grids using random forest model. Prog. Geogr..

